# Editorial and Miscellaneous

**Published:** 1863-08

**Authors:** 


### EDITORIAL AND MISCELLANEOUS.

*A Treatise on Hygiene with Special Reference to the Military Service.* By WILLIAM A. HAMMOND, M. D., SURGEON GENERAL, U. S. ARMY; Fellow of the College of Physicians of Philadelphia, Member of the Philad. Pathol. Soc.; of the Acad. Nat. Sci.; of the Am. Philosoph. Soc.; Hon. Corresp. Memb. of the Brit. Med. Assoc.; Member of the Verein fur Gemeinschaftliche Arbeiten zur Forderung der Wissenschaftlichen Heilkunde; late Prof. of Anatomy and Physiology in the University of Maryland; late Surgeon and Lecturer on

Clinical Surgery at the Baltimore Infirmary, &c., &c. Philadelphia: J. B. Lippincott & Co., 1863. pp. 604. $5.00.

We welcome this work with peculiar satisfaction. Primarily because it is a timely work—one which the present exigencies of our country demand, secondarily because it is a valuable contribution to general science. The Surgeon General of the United States' army, occupying a position of responsibility inferior to none of which the annals of history afford any record, and involved in duties unparalleled, has found it necessary to undertake a task of the most vast importance. Recognizing the grand principle that the prevention of disease is a work, if possible, more important than its cure, and keenly alive to the fact that sanitary science has thus far been less cultivated than therapeutics, he has in addition to his onerous public duties undertaken a task from which a private, otherwise unoccupied, scholar might well shrink. The proof of this proposition is found in the fact that there is scarcely an attempt at an adequate treatise upon the subject in the English or indeed in any other language. Here and there may be found a tiny manual, or diminutive monograph, but not a single work at all commensurate with the subject.

When we reflect that in addition to his other official duties Surgeon General Hammond has within the last year organized military hospitals, unsurpassed in perfection of details by any in the world, numbering more than *one hundred thousand* beds, and that all along a military line of many thousand miles his wonderful administrative and executive abilities have reached, with a perfection of success which has wrung from his bitterest enemies the (to them, humiliating) confession that "THE MEDICAL AND HOSPITAL ORGANIZATION OF THE SERVICE IS MOST THOROUGH AND COMPLETE"—we may well marvel at the zeal, professional *esprit de corps*, and better than all, high-toned, self-sacrificing patriotism which could attempt an arduous labor like that which has culminated in the work now before us.

Since writing the article of last month, defending General

Hammond from the violent, malignant and unprofessional attacks made upon him in certain quarters, in consequence of his famous "Order No. 6," we have taken occasion to review his connection with the medical department of the army as its official head. We have carefully read every order or circular from the department since he was placed in command, and made ourselves familiar, so far as possible, with the practical workings, and we do not hesitate in publishing this our conviction, that more than Baron Larrey was to the first Napoleon, what Washington was to the Revolution, and what Grant thus far appears to the army of the Mississippi, has been Hammond to the medical staff of the grand army of the United States.

Need we undertake to express the unmitigated disgust, abhorrence and contempt, we feel for the *animus* which carried through the following resolution in a medical society not far from Ohio?

"*Resolved*, 5th, That Drs. Jno. A. Murphy, G. C. E. Weber, and A. Metz be a committee instructed to report to the American Medical Association that this Society demands the speedy trial and expulsion of Dr. Wm. A. Hammond, for the gross injustice done the profession by his foolish and quackish order No. 6."

We here put down the prophesy that there is not an individual who voted for that resolution, but that in a twelvemonth (if not already) will be ashamed of it, and in five years will deny that he ever voted for it, or was even present at the meeting upon whose records it has become "damned to everlasting fame."—The book before us is sufficient answer to all such narrow-minded, illiberal twaddle. In the words of a dignified and learned contemporary, the veteran *Boston Medical Journal*:

"No better proof of the fitness of the present Surgeon General of the United States for the high and responsible office which he fills could be asked than the volume before us. It will take its place at once among the standard works on the subject. Any one conversant with the philosophical and inquiring mind of the author will readily understand that he is to be found among the most influential class of the profession at the present day, who are disposed to question pretty closely the claims of drugs to virtues which too often prove to be merely traditional, while they are ever on the alert to detect those causes which produce and perpetuate disease, and depend more upon an accurate knowledge of the laws of nature in treating it than upon any specific medication. His recent exclusion of the two powerful agents, calomel and tartarized antimony, from the medical supply table of the United States' army, at once shows his confidence in the power of such methods of treatment, and his indifference to the criticisms of the routinists."

Yet Dr. Hammond is not indifferent or sceptical as to the efficiency of proper medication in the treatment of disease, he only urges that "the curative influences of hygienic measures have been too much neglected, and that drugs, the traditional actions of which have been positively disproved by physiological and chemical researches, as well as by the soundest deductions from pathology, are too frequently administered through a strict adherence to the routine which hinders the development of medical science, and cramps the powers of those who labor for its advancement."

The object of the book is to elucidate the principal facts which bear upon the hygienic conditions of man in causing, preventing and curing disease. He urges that, in the military service especially, the very salvation of an army often depends upon the knowledge and application of hygienic principles by those who have the medical superintendence.

It is not pretended that the volume is complete—the circumstances of its publication rendered that impossible—that which has been written is of the imperative practical sort, and he who understands it will not fail to carry his own reflections once thrown in the right direction to other points of great importance. The author defers to a second edition a more complete fulfillment of his allotted task.

Our readers are aware that under the sagacious and comprehensive direction of the Surgeon General an immense amount of material is being collected in the Army Medical Museum, and in the bureaus of the department—an amount unprecedented in the annals of military medicine and surgery, and more even than is contained in the published medical records of all the armies of the world,—and that, by-and-by, this immense accumulation of matter is to be systematized, and thus form a body of scientific medical and surgical truth the like of which the world has never known.

We cannot forbear to quote the concluding passages of Dr. Hammond's preface:

"It is only by yielding our opinions to the age in which we live, when every science bearing upon medicine is being developed by the labors of thousands of investigators, that we can claim the right to be regarded as wise physicians seeking only the good of those committed to our charge, rather than our own personal advantage. In science we believe nothing until it is proven, and even then we should be ready to forsake our most cherished doctrines when the evidence of their instability is forthcoming. If, therefore, I have been positive in the expression of opinions which are at variance with those held by others, it is only because I *now* believe them to be correct. To morrow I may renounce them all.

"But even in my positiveness, I hope I have not forgotten the proprieties of life, or the forbearance and courtesy which should prevail in all discussions, especially those of a scientific character."

Which gentlemanly as well as scholarly ideas we commend to the Ohio committee on Expulsion.

But *revenons à nos moutons*———

The book is divided into three sections. The first section, of two chapters, discusses the general and special qualifications and disqualifications of recruits. The second section, in ten chapters, discusses the agents inherent in the organism which affect the Hygienic condition of man, i.e. Race, General and Particular Temperaments, Idiosyncrasy, Age, Sex, Hereditary Tendency, Habit, Morbid Habits, Constitution.

The third section, of twenty nine chapters, treats of Agents External to the Organism which act upon the Health of Man, under which are noticed the Atmosphere with its essential and accidental constituents, Temperature, Light, Electricity, Water, Soil, Locality, Climate, Acclimation, Habitations, Hospitals, Principles of Hospital Construction, Field Hospitals, Lighting of Hospitals, Heating of do., Ventilation of do., Barracks, Camps, Food, Alimentary Principles, Physiological and Sanitary Relations of Food, Animal Compound Aliments, Vegetable do., Accessory Food, Alimentation of the Soldier, Clothing, Hygienic Relations of Clothing with the several parts of the Body. *Index.*

We have not space or time for an elaborate review of the author's views and statements upon these important topics. They should be thoroughly and carefully studied and digested —and the student or practitioner will not fail to arise from the perusal with more enlarged and comprehensive ideas of the physician's true sphere, and at the same time, with far more clear and practical notions as to his every day, and every hour, duties.

We chronicle this book, emanating from the source which it does, as a distinct milestone of medical progress. Well may its author laugh to scorn the puny attempts of his detractors, whether single or associated.

---

*True Function of the Round Ligament of the Hip Joint.* —John Struthers, M. D., &c., Lecturer on Anatomy in the Edinburgh School of Medicine, in the London *Lancet* demonstrates the true function of the round ligament of the hip joint. He observes in concluding his article:

"Whatever may be the explanation of the direction of the different dislocations, it is evident that the natural tendency is for the bone to throw itself out of the socket forwards. Now to prevent this, there are two strong ligaments. In the extended position, as in standing with the toes turned more or less out, it is checked by the whole front of the capsular ligament, including the entire ilio-femoral band. But by flexion the front of the capsule is relaxed, allowing the outward rotation to go farther, until it is checked by the round ligament, and by the outer part of the ilio-femoral band. The limb in this position when it is lifted and advanced in walking, or in stepping up with the toes everted, in sitting with the knees apart, or with one leg laid across the other knee, or in the tailor posture, or on horseback. In all these and similar positions the hip joint is flexed and rotated outwards, and the ligament is called into play to prevent the ball starting forward from the socket."

---

*Radical Cure of Hernia.*—Mr. Wood's operation is commended in the same journal as among the best in vogue. It is comparatively simple, and certainly in point of danger is an absolute contrast to the formidable operation involving the considerable external incision practiced in cases of large herniæ. The operation in one clinical case is reported as follows. The patient being under chloroform: "The finger being introduced in the external ring, and as far as possible beneath the edge of the conjoined tendon, one of Mr. Wood's needles, made for the purpose, was introduced through the skin over the point of the finger, pushed through the conjoined tendon, being then guided by the finger through the internal pillar of the external ring before it pierced the skin of the scrotum. Another needle, then introduced through this lower puncture and external ring, was pushed through Poupart's ligament, and out at the upper puncture by the side of the first needle. The needles, having a sort of eye twisted in them, were thus able to be locked firmly together, so as to draw the walls of the inguinal canal and the pillars of the ring together. The needles were fastened down with plaster and a pad and bandage. Needles removed in 14 days. The cure was complete."

---

*Fractured Thigh.*—T. WILKINS, M. D., late of the U. S. A., now of Greenville, Bond Co., Ill., writes us that in cases of fractured thigh, where more elaborate apparatus is not at hand, he has found the following very convenient: A stout frame, a little longer than the patient, and three feet in width. Strong canvass is securely fastened to this. A round hole is cut in the cloth at the region of the nates, and bound around the edge. The patient is laid upon this canvass, and then the whole upon the bed. He is allowed to remain upon it all the time, except when the bowels are to move, when the frame can be lifted off from the bed and supported upon chairs or stools, the receiving vessel being placed under the opening. The duty being performed, all can readily be replaced upon the bed, with not the slightest danger of displacing the fractured bones.

---

*Prof. Bedford's Diseases of Women and Children—Erratum.*—In accordance with the frequently expressed wish of subscribers, we undertook last month to append to some of our book notices the respective prices, Not being furnished with that of the one at the head of this notice we copied from the Cincinnati *Lancet* the price as $2.00. This it appears was a typographical error, as a moment's reflection would have shown us. A book of that size could never be furnished at that price, in war times at least. As we stand corrected by a note from the publishers, Wm. Wood & Co., New York City, the price is $3.50.

---

*Obituary.*—Charles Hartman, M. D., of Cleveland, Ohio, well known as former Collaborator of the Cincinnati *Lancet & Observer*, fell upon the battle field of Chancellorville, May 3d. He was, we believe, at the time, a surgeon of volunteers. His death will be much regretted as that of an educated, accomplished and high-minded physician.

Samuel A. Cartwright, M. D., near Jackson, Miss., Æ.72. Well known for his peculiar treatment of Asiatic Cholera, his papers on the Sugar House Cure of Consumption, Experiments on the Aligator, &c., &c. Dr. Cartwright was long a resident of Natchez, and afterwards removed to New Orleans, whence he communicated to the Northern medical press copiously. We are not informed whether he was connected with the medical staff of the Confederate army, but presume, from his advanced age and kindly feelings, in past time, toward the North, that he was not. His death will be generally regretted, both North and South, as a genuine medical gentleman and scholar of the old school.

*Sea Sickness.*—℞. Hydrochlor. Acid. Dilut., ʒ ij; Acid Nitric Dilut., ʒ j; Acid Hydrocyan., gtt. xvj; Aquæ, ℥ viij; M. S. Two tablespoonfuls every three or four hours.—*Drug. Circ.*

"*Ludlam's Specific.*"—℞. Ext. Krameriæ, ℥ ij; Pulv. Aluminis, ʒ j; P. Cubeb., ℥ j; Bal. Copaib., ℥ ij; Magnes. Carb. q. s. M. f. massa.—*Id.*

*Camphor Ice.*—℞. Ol. Amygd. Expr., Aq. Rosæ, *aa* lb., j; Ceræ Albæ, Spermaceti, *aa* ℥ j; Camphoræ, ℥ ij; Ol. Rosmarinæ, ʒ j; M. S. Artem.—*Id.*

*Vinum Anti-Leucorrhoicum.* (HAGER.)—℞. Cort. Cinchonæ, 6 parts; Tinct. Cinnamomi, 2 parts; Rad. Ratantiæ, 20 parts; Catechu, 5; Sacchari, 100; Vini Rhenani, 10,000; Alcohol, dilut., 100. Macerate together, express and filter. S. Twice daily a wineglassful.—*Id.*

*Formula for Use of Bromine.* (Prof. J. L. SMITH.)—℞. Bromine, 1 troy ounce; Bromide of Potassium, 160 grs.; distilled water, q. s., to make four fluid ounces of the whole mixture. Dissolve the Bromide of Potassium in about two fluid ounces of the water in an eight ounce bottle, then add the Bromine, agitate gently until the solution is complete, then add water enough to bring the whole to four fluid ounces. This mixture forms a very dark red solution, evolving strong fumes of Bromine, and readily soluble in any additional quantity of water.—*Am. Jour. Med. Sci.*

*Glycerole of Iodine.*—℞. Glycerinæ, 2 parts; Potassii Iodid., 1 part; Iodinii, 1 part. M. f. sol.—*Drug. Circ.*

*Death from Inhalation of Nitric Acid Vapor.*—Two cases of death are reported in Edinburgh from accidentally inhaling largely the fumes of Nitric Acid. In one case the first symptoms of poisonous effect came on in an hour, with death at ten hours after. In the other not until three hours after the inhalation, and death in about thirty-six hours.

*Fatality of Cholera in Malabar.*—The mortality among such of the natives as were treated in hospital on the outbreak of cholera in 1859–60 was 66 per cent.; that of cases registered throughout the district no less than 93 per cent.—*London Lancet.*

*Oxygen Gas.*—At the last sitting of the Academy of Sciences of Munich, Baron Liebig made a very interesting communication relative to some experiments made with a new apparatus—manufactured chiefly at the expense of the King of Bavaria—for detecting the existence and measuring the quantity of oxygen in various bodies. The experiments, Baron Liebig stated, had proved clearly that oxygen is not only evolved from the atmosphere by plants, but also in tolerably large quantities by decomposition of water in the body of flesh-eating animals. Baron Liebig thinks that the knowledge of this fact will throw quite a new light on the hitherto but imperfectly understood processes of nutrition and digestion.—*Id.*

---

*Perjury by a Surgeon.*—At the assizes at Liverpool, on the 28th ult., Evan Thomas, a surgeon, of Manchester, was tried for perjury. The offence had been committed in a deposition made before the Manchester City Coroner, at an inquest held on the body of a widow named Bell. Mrs. Bell had come from Cockermouth, and was met by the prisoner at the railway station at Manchester. He conducted her to the Cathedral Hotel, and spent a few minutes with her there. She was then very well. Next morning she was very unwell, and Thomas visited her. When he entered the room he locked the door, and shortly afterwards came out saying that Mrs. Bell was dead. At the inquest on the body he swore that he had not known her before, that she had a tumor in the womb, and that death had been caused by epilepsy. Her friends at Cockermouth subsequently caused an inquiry to be made, when it came out that Thomas had known and written to the deceased some months previously. Another post-mortem examination was held, when it was discovered that there was no tumor in the womb, that the disease was pregnant, and that, although the appearances were not inconsistent with death from epilepsy, the prisoner had never examined the brain to ascertain if there were epileptic symptoms. The statement made by him previously was thus shown to be false, and he was indicted for perjury. He was found guilty, and sentenced to three months' imprisonment, the judge expressing his opinion that the prisoner had carried on an illicit amour with the deceased.—*Id.*

Was not this an attempt to procure abortion by some injecting apparatus, whereby air gained admission to the uterine sinuses? This would readily explain the coincidence, unfortunately, getting quite too common, of sudden death "shortly after" the suspected person entering the room of the unhappy victim.

---

*A New Work on Practice.*—It is rumored, and a little bird has whispered it to us from the air, that Prof. Austin Flint—whom not to know is to argue one's self unknown, and not to respect shows one incapable of appreciating great personal, literary and professional worth—is preparing for the press a new work on the Practice of Medicine. Such a work is the great want of the time, and we most fervently trust the rumor is well-founded. We scarcely know, on the Continent, one to whom such a task could be as fitly entrusted. Long familiarity with medical students, as a teacher, will enable him to write those things which students need to know; large, varied and most successful practical experience, together with most magnificent capabilities as a diagnostician, will render what he says authoritative with practitioners; whilst literary and professional success in authorship, already achieved by his classical treatise on Diseases of the Heart and other writings, guarantee that the book when written will immediately take the first rank as the standard text-book, wherever the English language is read.

We have no means of knowing when the publication is to take place, and can only say that every day it is delayed is a misfortune to the profession.

Conservative Medicine is now a fixed fact, and the invalided grumblers, the fossils, and stand-stills, must fall into the rear of the grand army, with the baggage-wagons and the wounded. A great progressive science, now developing with most marvellous rapidity, must not be repressed and tied down by exploded ideas and the rotten ropes of effete dogmatism.

Looking across the Atlantic, the scattered waifs which have floated to us bearing the impress of THOMAS K. CHAMBERS, lead us to hope that before long the profession may have from his pen also, a systematic treatise, which we know they "will not willingly let die."

---

*Editorial Change.*—We regret to notice that Dr. R. J. Levis has retired from his connection with the *Philadelphia Reporter*, having entered the army as a Surgeon of Volunteers.

Dr. O. C. Gibbs had sometime previously discontinued his communications to the same journal, but from what cause we are not informed. These withdrawals are to be the more regretted as they have notably impaired the tone of the editorial columns of that medical weekly. Several of the recent numbers of our beloved Philadelphia contemporary disclose an editorial biliousness which would seem to indicate still further purging.

Briefly—Dear *Reporter*—do you not think that several of the loose evacuations of insinuation and imputation into which you have recently relaxed, would have been ameliorated by the simple remembrance, not of the conventional courtesies of journalism, but of what was due to yourself as a member of a, supposed, gentlemanly profession?

---

### ILLINOIS STATE MEDICAL SOCIETY.

The Special Committee on *Diseases of the Eye,* appointed at the last meeting of this Society, earnestly request all members of the association and of the profession generally, to aid in accomplishing the object for which the Committee was appointed.

Although the Committee has previously reported upon the causes of Conjunctival Inflammations as they prevail at the West, it is desirable to collect more facts in reference to the causes and prevention of this disease.

The Committee would suggest that all members, who have observed the progress of *Epidemic Conjunctivitis,* contribute such facts as may demonstrate to what extent the disease is influenced by dryness of the atmosphere, season of the year, dust from trees and plants, want of care as regards food and exposure, malaria, etc.

The history of interesting cases of any form of Ophthalmic disease is urgently solicited.

It is important that all communications be forwarded to the address of the subscriber previous to April 1, 1864.

E. L. HOLMES, M. D.,
Box 2175. Special Committee on Diseases of the Eye.

